# A140 DELAYED BLEEDING POST-ENDOSCOPIC AMPULLECTOMY FOR AMPULLARY ADENOMAS: INCIDENCE, RISK FACTORS AND MANAGEMENT OF DELAYED BLEEDING

**DOI:** 10.1093/jcag/gwad061.140

**Published:** 2024-02-14

**Authors:** K Pawlak, K Khalaf, S Gupta, D Tham, J Mosko, G May, N Calo

**Affiliations:** St Michael's Hospital, Toronto, ON, Canada; Division of Gastroenterology, St Michael's Hospital, Toronto, ON, Canada; Therapeutic Endoscopy, St Michael's Hospital, Toronto, ON, Canada; Faculty of Health Sciences, McMaster University Faculty of Health Sciences, Hamilton, ON, Canada; St Michael's Hospital, Toronto, ON, Canada; Division of Gastroenterology, St Michael's Hospital, Toronto, ON, Canada; St Michael's Hospital, Toronto, ON, Canada

## Abstract

**Background:**

The duodenal tumors of major papilla account 10% of all peri-ampullary lesions, and the majority represent adenomas, carrying malignant potential through the well-known adenoma–carcinoma sequence ([i]). Historically, surgical resection was the standard of care, but it is associated with significant risk of complications (44.7%) ([ii]). Hence, endoscopic ampullectomy became the treatment modality for selected cases. Despite the significantly lower rate of adverse events, pancreatitis and bleeding occurs in up to 25% of patients ([iii]). The rate of bleeding may be even higher, depending on periampullary lesion size and type. Factors related to delayed bleeding are poorly understood.

**Aims:**

The aim of our study was to determine predicting factors for delayed post-ampullectomy bleeding.

**Methods:**

We conducted a single-center retrospective study over 13 years (2010-2023). All patients who underwent an endoscopic ampullectomy were analyzed. The primary endpoint was the incidence of delayed bleeding, which was defined as a post-procedural bleeding that necessitated either a blood transfusion, ICU admission or re-intervention. Secondary outcomes included risk factors for delayed bleeding, management, and other adverse events.

**Results:**

113 patients underwent endoscopic papillectomy [mean age 66.2 ± 12.2 years; male gender 51 (45.1%)]. Mean lesion size was 27.0 ± 14.3 mm and mean procedure duration was 62.8 ± 35.6 minutes.

There were 24 cases of delayed bleeding (21.2%). Of these, 6 (25%) required repeat endoscopic intervention. The average length of hospital was longer in those experiencing a delayed bleed (8.6 ± 4.9 vs 4.8 ± 2.4 days, Pampersand:003C0.001).

By univariable logistic regression, the odds of delayed bleeding were greater in those with hypertension (OR 3.8, 95%CI 1.4-10.3, P=0.008) or an INR ≥ 1.2 (OR 13.3, 95%CI 3.0-58.3, P=0.001). A multivariable logistic regression analysis revealed that INR≥ 1.2 predicted delayed bleeding, with an OR of 16.1 (95%CI 3.0-85.4, P=0.001). Other adverse events included perforation (n=7, 6.3%) and pancreatitis (n=19, 16.8%). There were no deaths.

**Conclusions:**

Post-ampullectomy bleeding is a common adverse event in patients undergoing ampullectomy leading to more prolonged hospital stay. History of hypertension and elevated INR above 1.2 might be related to delayed post-ampullectomy bleeding. Additional strategies to reduce post-ampullectomy bleeding should be explored.

**Funding Agencies:**

None

